# Differential Diagnosis of an Elderly Manic-Depressive Patient with Depersonalization and Other Symptoms

**DOI:** 10.1155/2016/1454781

**Published:** 2016-05-18

**Authors:** Shigehiro Ogata, Yu Itohiya, Yuri Sakamoto, Yuki Sato, Yudai Suyama, Hidenori Atsuta, Ken Iwata

**Affiliations:** ^1^Department of Biochemistry and Cellular Biology, National Institute of Neuroscience, National Center of Neurology and Psychiatry, 4-1-1 Ogahahigashi, Kodaira, Tokyo 187-8502, Japan; ^2^Department of Psychiatry, Toshima Hospital, 33-1 Sasae, Itabashi-ku, Tokyo 173-0015, Japan; ^3^Department of Neuropsychiatry, Tokyo Metropolitan Hiroo Hospital, 2-34-10 Ebisu, Shibuya-ku, Tokyo 150-0013, Japan

## Abstract

The case study of an elderly man having persecutory delusions and bizarre complaints at the first psychiatric interview is reported. The patient complained: “I have no sense of time” and “I have no sense of money.” He refused nursing care. He had delusions centered on himself including that of his own death, which were difficult to diagnose but suggested the possibility of Cotard's syndrome. We assumed that the man was depressed and treated him for depression. However, as a result of this treatment he became temporarily manic but finally recovered completely. After his recovery, we learnt the patient's past history of hospitalization for psychiatric problems, and based on that history he was diagnosed as suffering from a bipolar I disorder. The lack of typical symptoms of depression and the remarkable depersonalization and derealization in this patient made it difficult to infer a depressive state. Nevertheless, being attentive to his strange feelings related to the flow of time would have helped us to make an accurate diagnosis earlier.

## 1. Introduction

Geriatric depression has not been sufficiently investigated to date. One reason for this could be that elderly patients compared to younger patients complain less often about depressive moods and more often emphasize somatic symptoms [1, 2]. Depression might also be accompanied by temporarily cognitive impairments that are often referred to as pseudodementia that can imitate frank dementia [2]. Therefore, when making a diagnosis of depression in elderly patients, it is important to consider frank dementia as a possible differential diagnosis.

An elderly inpatient attending our hospital was diagnosed with Cotard's syndrome, which is a rare disorder, in which the central symptoms are nihilistic delusions [3, 4]. We treated this patient using modified electroconvulsive therapy (mECT), which resulted in a manic episode. However, the psychiatric history of this patient, which we could only obtain after mECT, might have resulted in diagnosing the patient with bipolar depression with psychotic symptoms.

Typical depressive symptoms, such as depressive mood, were not observed in this patient. Rather, the patient made many strikingly odd complaints that could be explained as depersonalization and derealization. Therefore, we considered the differential diagnoses of neuropsychiatric diseases, such as delusions of guilt, or Cotard's syndrome, both of which developed during the course of his illness. This made us focus on the likelihood that the patient was suffering from a depressive state and his complaints about time or, more correctly, his feeling about the flow of time was indicative of depression. The case study of this patient is presented.

## 2. Case-Presentation

A 70-year-old man, Mr. I, had been restless at home because of a false belief that police would arrest his family. His family made him undergo a medical examination by a general practitioner in our hospital. His vital sign and physical examination, including neurological signs, were normal. Moreover, his blood test showed no abnormalities. The doctor prescribed an antipsychotic, 25 mg of quetiapine before sleep. The next day he was referred to the psychiatric department for evaluation.

The patient had worked as an accountant in a travel company for 30 years. He had at times lost over several million yen on stock investments and horse racing. However, until the current episode, his family had been unaware of these behaviors or his mood instabilities. In his midfifties, the patient was fired when his company downsized. Thereafter, he had found new employment as a security guard and had worked for over ten years in this job. Approximately one month previous to the consultation, he had stopped work for personal reasons and had requested to be retired, because of his long continuing hearing problems. Soon after retirement, the patient began to complain that he was losing his memory.

During the medical examination, the patient looked very perplexed. The examination indicated that his vital signs were normal. He could remember that he had come to our hospital the day before. However, he could neither remember how he had come nor remember how he went back home, nor recall what he had done at home the day before. He repeatedly said that he has lost his memory. He also asked repeatedly for the time and complained that he felt as if time had passed very fast. He could not respond correctly to questions about the day of the week nor state the current year and seemed disoriented. In addition, he mentioned money. He had lost the sense of handling money and questioned the real value of a yen. He did not respond to questions about delusional ideas. He could respond to certain requests, such as grasping things with his hand, when asked to do so.

We suggested getting admitted to hospital for further examination and treatment. The patient's electroencephalogram and magnetic resonance imaging (MRI) indicated no abnormalities. The color, osmotic pressure, cell numbers, glucose, and lactate dehydrogenase (LDH) of his spinal fluid proved to be normal. In his regular blood test, creatinine was temporarily elevated up to 1.3 mg/dL, because he rejected meals during the early period of his hospitalization (as seen below) and dehydration emerged. However, after the treatment progressed, creatinine returned normal. We prescribed 50 mg of an antipsychotic, quetiapine, before sleep. He ate well on the first day of hospitalization and continued to do so, but his complaints did not change. Moreover, he continued to walk in the ward corridors, he was occasionally confused, and sometimes he had a grin. He told us, “I know that I am hospitalized now, but I don't know what date it is, or how long I have been in hospital” and that “days have gone by without me noticing.” He said, “time passes very fast, I feel no sense of time, and it seems as if time has stopped.” He had a calendar in his pocket and often referred to it to confirm the date. In addition, he stated, “I feel as if I didn't wash my body.” He also mentioned losing money.

Seven days after admission, the patient refused to bathe or undergo any medical examinations. Eleven days after admission, he covered his ears with his hands. It seemed that he was having auditory hallucinations, but he could not describe their content. However, he described a nihilistic delusion while stamping his foot and stating, “I am dead.” He also described other delusions such as “I have become a stone,” “my heart has stopped,” “I can go 300,000 light-years ahead,” “I have destroyed all mankind,” or “loans have accumulated to an astronomically large amount.” He sometimes grinned and was in a mild stupor. Then, he began to refuse food and resisted nursing care. The antipsychotic medications, risperidone, olanzapine, and quetiapine, were sequentially prescribed for his delusions and hallucinations; however, his condition remained unchanged. He denied having a depressed mood. The tricyclic antidepressant clomipramine was administered via intravenous drip, because it was possible that his symptoms were indicative of psychotic depression. However, the antidepressant had no effect on his condition.

mECT was started on the 35th day after admission. The patient's rejective attitude was alleviated following the second administration of ECT, and he began to eat. He also became well oriented and stopped talking about time or money. After the fifth mECT, his mood became highly elevated and irritated. He talked loudly and could not help talking indiscriminately and loudly to other patients. As a result, we suspected a hypomanic condition and stopped further mECT treatment and added sodium valproate, which is a mood stabilizer to the prescribed antipsychotic, 15 mg of olanzapine per day. Sodium valproate was maximized at 1000 mg per day and, approximately two weeks later, his mental state became normal. Then, olanzapine was tapered to 10 mg/day. At this time, he could neither remember his delusions nor remember what he had said just after hospitalization.

Following improvement, further information was obtained. The patient had no family history of psychiatric disorders. We also discovered that this patient had once in his adolescence been admitted to a psychiatric hospital for psychiatric problems. He stated that at the time he had a highly elevated mood and had felt that he had superpowers. He also said that he had been treated with ECT, but he did not tell us his diagnosis. According to him, he had never experienced depression. He was discharged from hospital 90 days after admission.

## 3. Discussion

The patient in this case study was diagnosed with a bipolar I disorder and a current episode of severe depression with psychotic symptoms. The course of depression in the patient was acute and severe. Approximately seven days after hospitalization, symptoms of Cotard's syndrome, originally described by June Cotard in an anxious, melancholic patient, include nihilistic delusions and délire d'énormité [4–6] emerged. In this case, a strong rejection of food and all nursing care was remarkably observed too. Cotard's syndrome is rarely observed in patients with depression. Losing money was a strong regret for this patient and he made bizarre statements that indicated delusions of poverty and delusions of guilt. From these observations, it was concluded that the patient was suffering from depression, and, based on this assumption, he was given clomipramine by injection, which had no effect on his condition. Therefore, we adopted mECT treatment. This treatment had a dramatic response on the patient, and he became hypomanic. There are no established guidelines about ECT-induced mania or hypomania, and it remains unclear whether this state should be treated with mood-stabilizing agents acutely or after ECT treatment [7]. One case report discussed the effectiveness of lithium against preventing switching manic episode during ECT [8], but it is still debated whether lithium medication is harmful during ECT or not. In addition, lithium has a low therapeutic index [9]. Our patient had temporally mild creatinine increase because of dehydration in the early period of the hospitalization. Therefore, in the hypomanic state, we decided to quit mECT treatment and give medication of sodium valproate.

After the patient improved, however, it turned out that he had been hospitalized for psychiatric problems during his adolescence. Judging from his statements and his prognosis, we considered that he should have been previously diagnosed with mania, and, therefore, we diagnosed him as suffering from bipolar disorder. It seems that this patient had experienced a manic episode followed by a depressive episode in the course of his life, with an interval of more than 50 years between the two episodes.

The diagnosis of a bipolar disorder is not very rare; however, the diagnosis was hard in the case of this patient, because it was difficult to discern the depressive state at the first medical examination, before the appearance of Cotard's syndrome. One reason for this failure is that we could not obtain an accurate medical history of the patient's illness, either from the patient himself, who was confused, or from his family. However, we want to emphasize that common symptoms of depression, such as depressive mood, were not conspicuous in this case. Moreover, at first, the patient's appetite was good, and he did not lie on bed during daytime. His way of walking around the ward might have been a sign of anxiety, but he did not express any anxiety.

The patient's state was mainly psychotic, but the delirium state must be considered too. Therefore nonorganic acute transient psychotic disorder or dementia, epilepsy, encephalitis, and other organic diseases, all of which might cause delirium, were included among the differential diagnoses. However, no characteristic results were obtained in physical, biological, or radiological examinations, and organic diseases were unlikely.

Rather noticeable about this patient were the peculiar feelings he had about time and his repeated questioning about the value of money he used for everyday things. He was also perplexed because he felt as if he had not washed himself even though he had actually washed. The former symptom was interpreted as derealization, and the latter as depersonalization. Depersonalization is an experience in which the individual has a sense of unreality and detachment from themselves. Patients with depersonalization complain of feeling a lack of ownership of their body and feelings of loss of agency and emotional numbing. On the other hand, derealization consists of alterations in perceptions about a person's surroundings, such that the sense of reality about the external world is lost. It has been suggested that derealization is one form of depersonalization [10, 11].

Depersonalization and derealization deserve further investigation. Psychiatrists such as Enoch and Trethowan, Weber, and Dietrich have suggested that these symptoms have a close relationship to Cotard's syndrome [3, 12, 13]. A well-known French psychiatrist, Séglas, who contributed to popularizing the term Cotard's syndrome, reported that the presence of depersonalization was the first step in the development of Cotard's syndrome [14]. This case study corroborates the idea of Séglas.

Nevertheless, it is difficult to establish or even narrow the differential diagnosis in cases of depersonalization and derealization, because these symptoms are seen in many different psychiatric illnesses such as posttraumatic stress disorder (PTSD), panic disorders, and unipolar depressive disorder [15]. Recently, the relationship with bipolar disorders was investigated to identify the extent and frequency that patients with bipolar disorder experienced depersonalization and derealization. Moreover, certain studies have suggested that dissociative symptoms including depersonalization and derealization are associated with bipolarity [16, 17]. However, these studies have only compared patients with unipolar depressive and bipolar disorders, which makes it difficult to conclude that patients with bipolar disorders experience depersonalization and derealization symptoms more often than patients with other psychiatric illnesses.

It is possible that complaint made by this patient at the initial hospitalization regarding time was the key for judging his depressive state. The patient felt very odd, particularly about the passage of time, alternatively feeling that the passage of time had nearly stopped or that it progressed very fast. Depressive patients often report alterations in the subjective experience of the passage of time [18], but this complaint seems to have nothing to do with the change in the basic mechanism of objective time perception [19]. Psychiatrists have discussed the subjective time experience. For example, Bschor compared time experience of depressive patients with that of healthy controls or that of manic patients. He concluded that depressive patients found the subjective time to flow more slowly [20]. Many researches reached the same results, but Ratcliffe reported that some depressive patient perceived two conflicting feelings about time. He felt like time went slowly but at the same time he felt like time was running out [21]. These complaints have been interpreted from a phenomenological perspective. Straus suggested that in melancholia the “immanent” or “ego-time” of the movement of life slows down or gets stuck, whereas the “transient” or “world-time” goes on and passes by [22]. Kuhs also suggested that melancholic patients found the universal time accelerated in comparison to their ego-time [23]. The explanations are applicable to this case study. It is possible that the patient perceived the flow of ego-time und universal time differently, as flowing very slowly and very fast, respectively. Had we considered these explanations more extensively at his hospitalization, they would have suggested that this patient's complaints were indicative of a melancholic state.

## References

[1] Sadock B. J., Sadock V. A. (2005). *Kaplan&Sadock's Comprehensive Textbook of Psychiatry*.

[2] Taylor W. D. (2014). Clinical practice. Depression in the elderly. *The New England Journal of Medicine*.

[3] Enoch M. D., Trethowan W. H. (1979). *Uncommon Psychiatric Syndromes*.

[4] Debruyne H., Audenaert K. (2012). Towards understanding Cotard's syndrome: an overview. *Neuropsychiatry*.

[5] Cotard J. (1880). Du délire hypochondriaque dans une forme grave de la mélancolie anxieuse. *Annales Médico-Psychologiques*.

[6] Cotard J. (1888). Du délire d'énormité. *Analles Médico-Psychologiques*.

[7] Do J. L., Arcand L., Narang P., Lippmann S. (2014). ECT-induced mania. *Innovations in Clinical Neuroscience*.

[8] DeQuardo J. R., Tandon R. (1988). Concurrent lithium therapy prevents ECT-induced switch to mania.. *Journal of Clinical Psychiatry*.

[9] Procyshyn R. M., Bezchlibnyk-Butler K. Z., Jeffries J. J. (2015). *Clinical Handbook of Psychotropic Drugs*.

[10] Sierra M., David A. S. (2011). Depersonalization: a selective impairment of self-awareness. *Consciousness and Cognition*.

[11] Mula M., Pini S., Preve M., Masini M., Giovannini I., Cassano G. B. (2009). Clinical correlates of depersonalization symptoms in patients with bipolar disorder. *Journal of Affective Disorders*.

[12] Weber A. (1938). *Über Nihilistischen Wahn und Depersonalisation*.

[13] Dietrich H. (1971). Analysis of a case of ‘delire des negations’ (Cotard) by a neurologist. *Nervenarzt*.

[14] Berrios G. E., Luque R. (1995). Cotard's delusion or syndrome?: A conceptual history. *Comprehensive Psychiatry*.

[15] Hunter E. C. M., Sierra M., David A. S. (2004). The epidemiology of depersonalisation and derealisation. A systematic review. *Social Psychiatry and Psychiatric Epidemiology*.

[16] Akiskal H. S., Maser J. D., Zeller P. J. (1995). Switching from ‘unipolar’ to bipolar II: an 11-year prospective study of clinical and temperamental predictors in 559 patients. *Archives of General Psychiatry*.

[17] Oedegaard K. J., Neckelmann D., Benazzi F., Syrstad V. E. G., Akiskal H. S., Fasmer O. B. (2008). Dissociative experiences differentiate bipolar-II from unipolar depressed patients: the mediating role of cyclothymia and the Type A behaviour speed and impatience subscale. *Journal of Affective Disorders*.

[18] Thönes S., Oberfeld D. (2015). Time perception in depression: a meta-analysis. *Journal of Affective Disorders*.

[19] Droit-Volet S. (2013). Time perception, emotions and mood disorders. *Journal of Physiology Paris*.

[20] Bschor T., Ising M., Bauer M. (2004). Time experience and time judgment in major depression, mania and healthy subjects. A controlled study of 93 subjects. *Acta Psychiatrica Scandinavica*.

[21] Ratcliffe M. (2012). Varieties of temporal experience in depression. *Journal of Medicine and Philosophy*.

[22] Straus E. (1928). Das Zeiterlebnis in der endogenen Depression und in der psychopathischen Verstimmung. *Monatsschrift für Psychiatrie und Neurologie*.

[23] Kuhs H. (1991). Time experience in melancholia: a comparison between findings based on phenomenology and experimental psychology. *Comprehensive Psychiatry*.

